# Printing special surface components for THz 2D and 3D imaging

**DOI:** 10.1038/s41598-020-77998-9

**Published:** 2020-11-30

**Authors:** Bo Yan, Zhigang Wang, Xing Zhao, Lie Lin, Xiaolei Wang, Cheng Gong, Weiwei Liu

**Affiliations:** 1 Institute of Modern Optics, Tianjin Key Laboratory of Micro-Scale Optical Information Science and Technology, Nankai University, Tianjin, 300350 China; 2 School of Electronic Engineering, University of Electronic Science and Technology, Chengdu, 611731 China

**Keywords:** Imaging techniques, Imaging and sensing

## Abstract

The paper reports an off-axis large focal depth THz imaging system which consists of three 3D printed special surface components (two aspherical mirrors and an axicon). Firstly, the optical design software is used to design and optimize the aspherical parabolic mirror. Secondly, the optimized mirror is prepared by a 3D printing and metal cladding method. Thirdly, a THz axicon is designed for generation of quasi-Bessel Beam and a new geometric theoretical model of oblique incident light for axicon is established. Finally, the imaging system based on the special surface components is constructed. Its maximum diffraction-free distance is about 60 mm, which is 6 times higher than the traditional system. To verify the effectiveness, THz two-dimensional imaging experiments and three-dimensional computed tomography experiment are carried out. The results are consistent with the design and calculations.

## Introduction

Recent years, terahertz (THz) wave shows tremendous potential in the fields of nondestructive testing, imaging and communications due to its excellent properties. Among them, the high-performance and customizable special surface components play a crucial role. Considering the longer wavelength of THz, the accuracy of the relevant components is on the order of microns. It is much lower than that of visible light components. Accordingly, it is feasible to implement high-performance THz components and system by 3D printing technology which has the advantages of simplicity, rapidity and flexibility.

Since the popularity of 3D printers, there have been many studies about 3D-printed THz components, such as waveguides^1–4^, lens^5,6^, and grating^7^. However, few studies on the design and implementation of off-axis aspheric parabolic mirrors are reported. Although it is difficult to process and manufacture, the aspheric can effectively correct wavefront aberration without adding more elements to the optical system, which reduces amount and size of the components and simplifies the system greatly^8^. Moreover, as a common optical element, the parabolic mirror has many fantastic optical properties, for example, it can focus incident light well and have almost no chromatic aberration in the accuracy range of Gauss optics^9^. Compared with lenses, the parabolic mirror can reduce energy loss in THz band effectively. Therefore, the combination of aspheric and off-axis parabolic mirrors will make it possible to build low-loss and high-performance THz imaging systems.

In addition, as one of the most famous structured beams, Bessel beams were proposed in 1987^10^. In 1992, G. Scott et al. reported a method of converting incident infrared radiation into quasi-Bessel beams using axicons^11^. In 2005, N. Trappe et al. in the National University of Ireland generated quasi-Bessel beams in the 0.1 THz^12^. In 2009, W. Dou et al. from Southeast University reported the generation of quasi-Bessel beams in the THz band by using a 32-layer binary axicon^13^. In 2015, J. Liu and his team from Huazhong University of Science and Technology used 3D-printed axicons to realize the generation of arbitrary-order quasi-Bessel beams^14^. Since transverse intensity distribution does not change with propagation distance, the Bessel beams exhibit non-diffracting property in free space, which can be utilized to extend the focal depth of imaging systems. It’s well known that THz wave is able to penetrate different types of nonpolar and nonmetallic materials to realize perspective imaging. However, the imaging depth is limited by the focal depth of THz optical components. Accordingly, THz large focal depth imaging based on axicons attracts increasing attentions. In 2011, Zhang et al. reported THz imaging in dielectric media with quasi-Bessel beams^15^. Since then, THz imaging technology based on axicons has been widely studied^16–19^.

In this paper, aiming at the off-axis large focal depth THz imaging based on printed special surface components, the design and fabrication of aspherical parabolic mirror and axicon are introduced. Besides, different from most models of normal incidence, a new geometric theoretical model of oblique incident light for axicon is established. After that, the off-axis THz imaging system is constructed and demonstrated. The focus depth reaches 60 mm, which is about 6 times higher than the traditional THz imaging system. It can be improved to achieve 2D or 3D imaging.

## Design and theory

**Figure 1:**
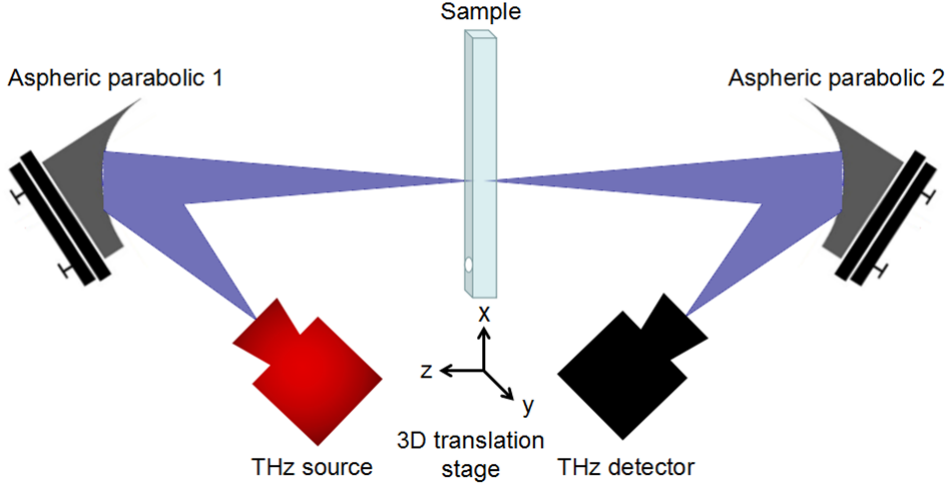
Schematic of the simplified off-axis THz imaging system without axicon.

The proposed THz imaging system consists of an axicon and two aspherical mirrors. According to the design principle from simple to complex, we first introduce the simplified aspherical imaging system without axicons. Its schematic diagram is shown in Fig. 1. The principle can be described as follows: The THz source emits the radiation, then it is reflected by the off-axis aspherical parabolic mirror 1; the imaged sample is placed at the focus position; the THz radiation passes through the sample and is reflected to the THz detector by the off-axis aspherical parabolic mirror 2. Driven by a translation system, the sample is scanned and its THz image can be acquired.

Off-axis parabolic mirrors, the core optical components of the system, are designed and optimized by the optical design software ZEMAX. The parabolic mirror 1 is the same as the mirror 2. Their initial design parameters are as follows: The wavelength is 3173um, the diameter is 50 mm, and the light turning angle is 60 degrees. The selection of surface type is the key to the parabolic mirror design. The standard surface is a common surface in most optical component designs. Its surface coordinate (z-coordinate) is given by:

1 $$ z = \frac{{cr^{2} }}{{1 - \sqrt {1 - (1 + k)c^{2} r^{2} } }} $$

where *c* is the curvature (the reciprocal of the radius), *r* is the radial coordinate in lens units and *k* is the conic constant. The curvature stands for the degree of curvature of the surface. When the curvature is infinite, the surface becomes a plane. The surface can be set to paraboloid, hyperboloid, elliptic surface, or sphere by conic constant. The conic constant is less than − 1 for hyperbolas, − 1 for parabolas, between − 1 and 0 for ellipses, 0 for spheres, and greater than 0 for oblate ellipsoids.

The difference between an odd asphere and a standard surface is the addition of a polynomial expansion of the deviation from a spherical (or aspheric described by a conic) surface. The odd asphere surface sag is given by:

2 $$ z = \frac{{cr^{2} }}{{1 - \sqrt {1 - (1 + k)c^{2} r^{2} } }} + \beta_{1} r^{1} + \beta_{2} r^{2} + \beta_{3} r^{3} + ... $$

Here, *c* is the curvature, *r* is the radial coordinate in lens units and *k* is the conic. *β*_i_ (i = 1,2,3,…) are the multi-order deviation parameters for odd-aspherical surface. We use the curvature, conic, first-order parameter *β*_1_, second-order parameter *β*_2_, and third-order parameter *β*_3_ as optimization variables to optimize the off-axis mirror. Moreover to compare the standard surface and aspheric surface, we optimized the two surfaces separately. The standard surface’s optimization variables are curvature and conic. Table 1 compares the key parameters of the optimized standard surface and the odd aspheric surface.

**Table 1:** Key parameters of the standard surface and the odd aspheric surface.

	*C *(curvature) (mm)	*k *(conic)	*β*_1_ (1th order)	*β*_2_ (2th order)	*β*_3_ (3th order)
Standard	− 117.8	− 0.687	None	None	None
Odd asphere	− 25.5	− 0.986	− 0.071	0.015	1.148E−005

**Figure 2:**
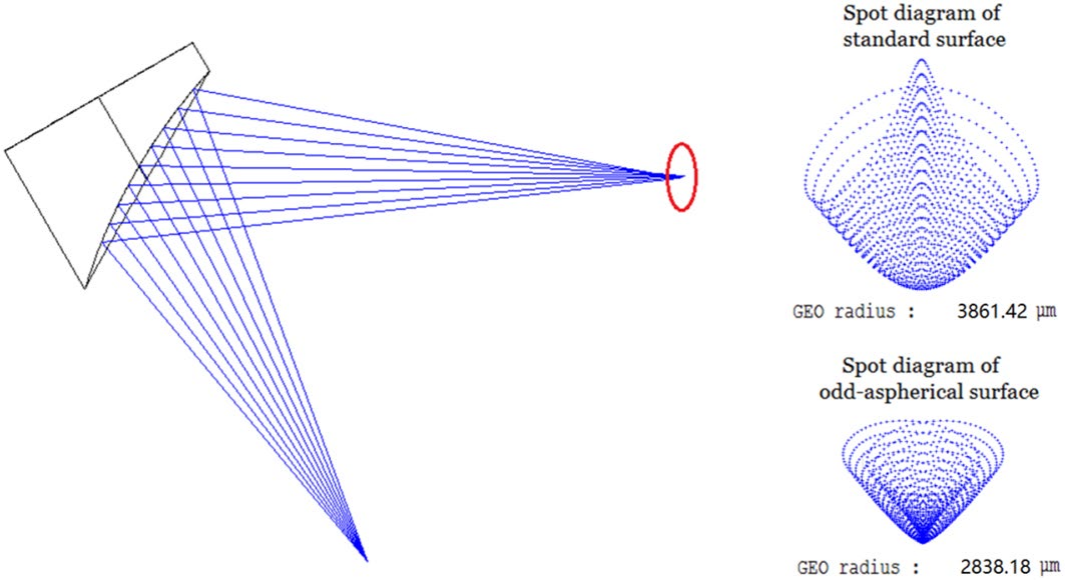
Comparison of focusing effect after optimization: aspheric surface and standard surface.

The optimization results are shown in Fig. 2. The focusing effect (spot diagram) at 193 mm is on the right side of the figure. It can be seen that the geometric radius of the focusing spot of aspheric surface can reach 2838.18 μm after optimization, while that of standard surface is about 3861.42 μm. Therefore, the aspheric surface system can focus the beam better and achieve a smaller scanning spot theoretically.

**Figure 3:**
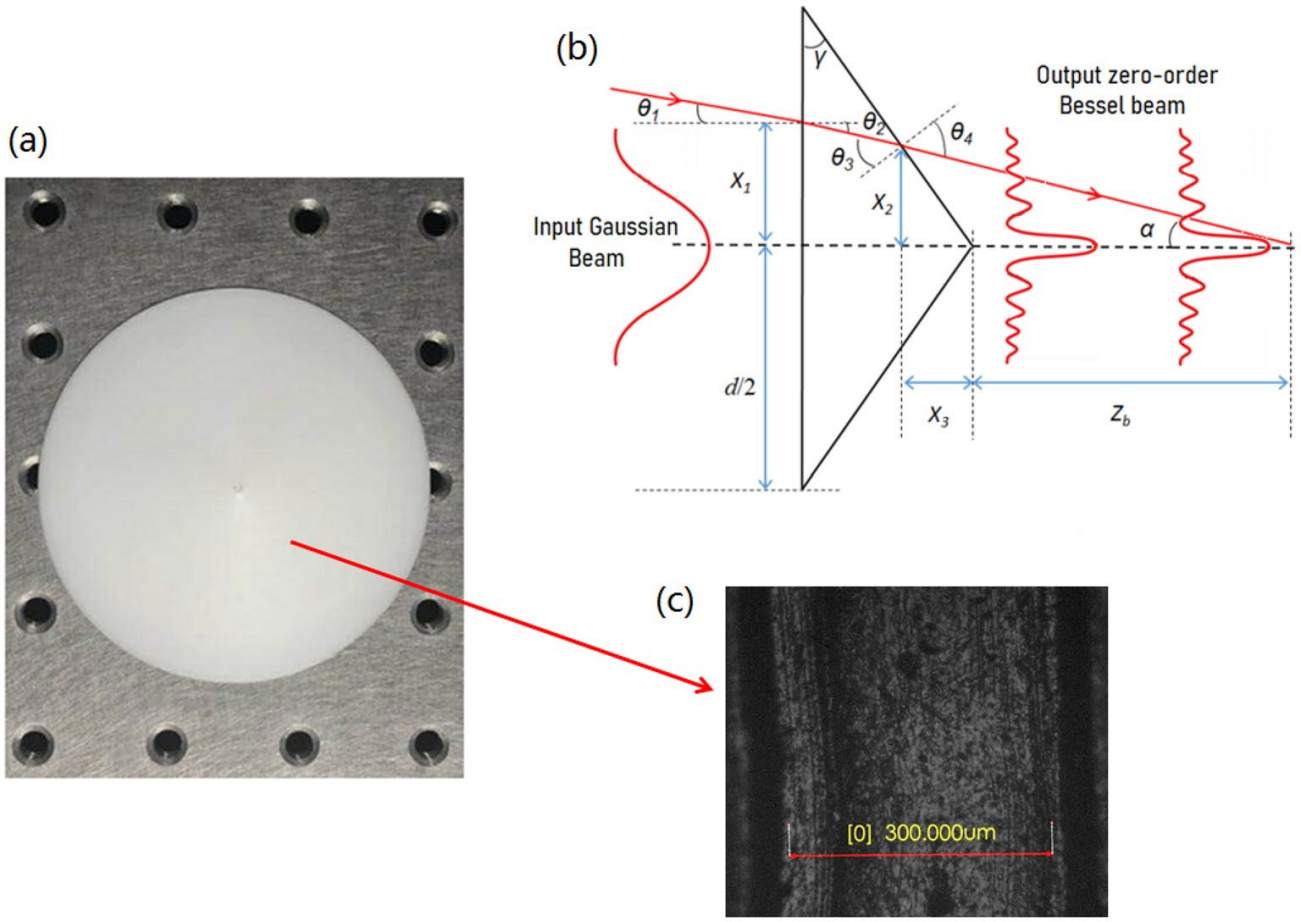
(**a**) Photo of 3D printed axicon; (**b**) schematic of light oblique incidence for axicon; (**c**) microscopic image of the specific area on the axicon surface.

Next, in order to generate “large focal depth” it’s necessary to realize the “non-diffracting” transmission characteristics of ideal Bessel beam. It allows the light beam to travel in the direction of infinite extension, carrying unlimited energy. It can be considered that the lateral electric field distribution is invariant along the axis. An ideal Bessel beam cannot be created in practice, but a quasi-Bessel beam over a finite range can be realized. In our system an “axicon” is designed and printed as shown in Fig. 3a, which can convert other types of light beams (such as Gaussian beams) into quasi-Bessel beams. Its schematic is shown in the Fig. 3b and the microscopic image of the specific area on the axicon surface is depicted in Fig. 3c. After passing through the axicon, the Gaussian beam is transformed into zero-order Bessel beam, which not only realizes the focus, but also propagates a long distance (*Z*_*b*_) with “non-diffraction”, which is far beyond the focal depth of the traditional Gaussian beam. Therefore, it is feasible to use axicon in the system to realize “large focal depth”.

As shown in Fig. 3, the 3D printed axicon’s bottom angle *γ* is 10°; the diameter *d* is 75 mm. The material is PLA (refractive index n = 1.6). In general, the light incident on the axicon can be regarded as approximately parallel. However, different from the parallel incident situation reported in most papers, the axicon in the off-axis system will face the case of oblique incident light. Therefore, a new geometric theoretical model is established to calculate the maximum diffraction-free distance *Z*_b_ as shown in Fig. 3b. According to the law of refraction and geometric relation, the angle *α* between the light passing through the axicon and the horizontal line can be calculated by:

3 $$ \left\{ {\begin{array}{*{20}l} {\alpha = \theta_{4} - \gamma } \hfill \\ {n_{0} \sin \theta_{1} = n\sin \theta_{2} } \hfill \\ {\theta_{3} = \theta_{2} + \gamma } \hfill \\ {n_{0} \sin \theta_{4} = n\sin \theta_{3} } \hfill \\ \end{array} } \right., $$

where *n*_0_ is the refractive Index of air; *n* stands for the refractive index of material of axicon; *γ* is the bottom angle. After obtaining the angle *α*, the maximum diffraction-free distance *Z*_b_ can be calculated by geometric relation. The relevant equations is expressed as

4 $$ \left\{ {\begin{array}{*{20}l} {z_{b} = - x_{3} + {{x{}_{2}} \mathord{\left/ {\vphantom {{x{}_{2}} {\tan \alpha }}} \right. \kern-\nulldelimiterspace} {\tan \alpha }}} \hfill \\ {x_{3} = x_{2} \tan \gamma } \hfill \\ {b = ({d \mathord{\left/ {\vphantom {d {2)\tan \gamma }}} \right. \kern-\nulldelimiterspace} {2)\tan \gamma }}} \hfill \\ {\tan \theta_{2} = {{(x_{1} - x_{2} )} \mathord{\left/ {\vphantom {{(x_{1} - x_{2} )} {(b - x_{3} )}}} \right. \kern-\nulldelimiterspace} {(b - x_{3} )}}} \hfill \\ \end{array} } \right.. $$

Here, the distance parameters (*x*_1_, *x*_2_, *x*_3_) and the angle parameters (*θ*_1_, *θ*_2_, *θ*_3_, *θ*_4_) are described in Fig. 3b.

## Experiments and results

**Figure 4:**
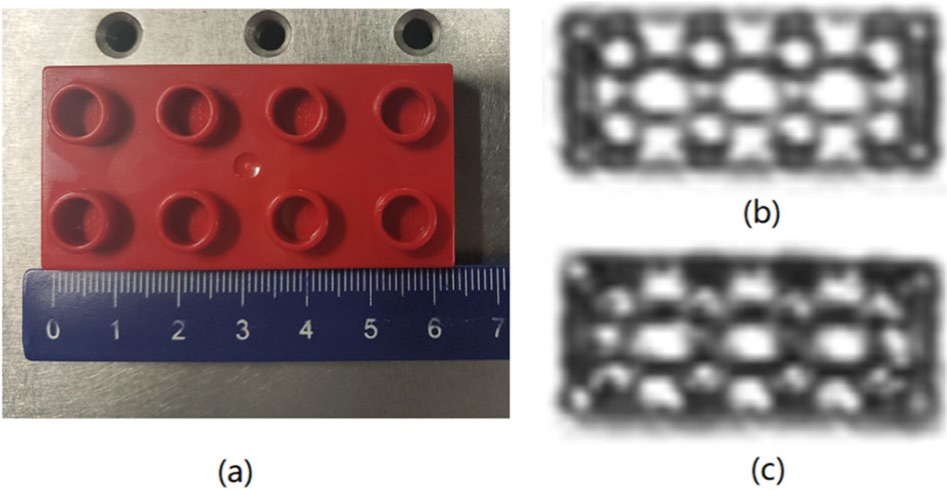
(**a**) the front view of a LEGO building block; (**b**) imaging result of the aspheric surface imaging system; (**c**) imaging result of the standard surface imaging system.

First of all, a comparison experiment based on standard surface imaging system and aspheric imaging system were carried out to evaluate the imaging effect of different surfaces. Schematic of the system is shown in Fig. 1. The imaging target in the experiment is a LEGO building block with a size of 63 mm × 32 mm as shown in Fig. 4a. It has 8 round holes on the front (4 on the top and 4 on the bottom, each hole is 9 mm in diameter), and three large round holes on the back (each hole is 14 mm in diameter). Figure 4b and c are the imaging results of the aspheric surface imaging system and standard surface imaging system, respectively. It can be seen that the terahertz radiation penetrates the LEGO building block; the 8 circular holes on the front and the 3 circular holes on the back are imaged. However, the aspherical surface system can distinguish the 8 round holes on the front and 3 holes on the back well. In contrast, although the standard surface imaging system can distinguish the 3 large circular holes on the back, the 8 circular holes on the front have poor definition and are difficult to distinguish effectively. Therefore, the aspheric surface has a better focusing spot, and the resolution is higher than that of the standard surface system.

**Figure 5:**
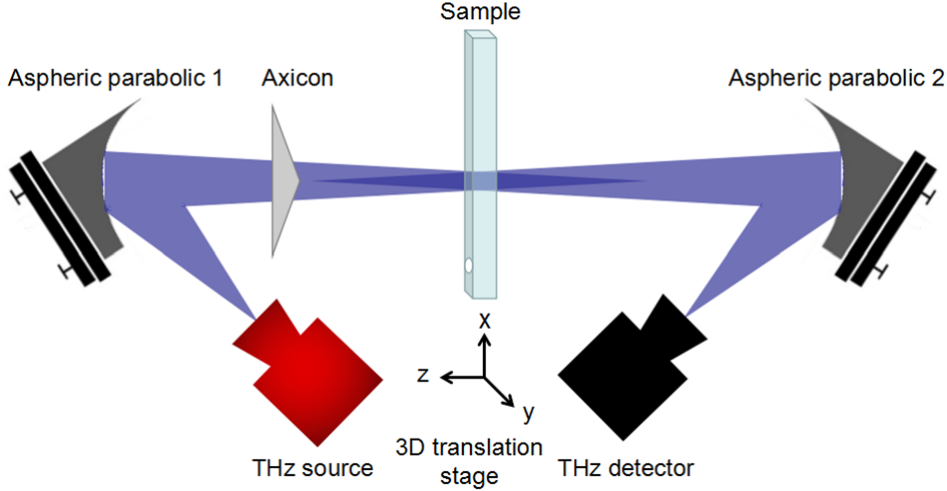
Schematic of the off-axis large focal depth THz imaging system.

Secondly, to extend focal depth of the aspherical imaging system the 3D printed axicon is added. Figure 5 describes the system’s schematic. The THz radiation is emitted by an IMPATT diode source (the frequency is 0.0945 THz and power is 80 mW), and reflected by the off-axis aspherical parabolic mirror 1; the axicon is placed about 44 mm behind the parabolic mirror 1 to produce quasi-Bessel beams and the sample is placed behind the axicon; the radiation passes through the sample and is reflected to the THz detector by the off-axis aspherical parabolic mirror 2. The distance between the axicon and sample is variable. The sample is fixed on a three-dimensional translation system and the THz image can be obtained by performing three-dimensional scan. It’s known that the axicon’s bottom angle *γ* is 10°; the diameter *d* is 75 mm, the distance between the axicon and the parabolic mirror1 is 44 mm and the oblique incidence angle *θ*_*1*_ is 6.43°. Therefore, according to the Eqs. (2) and (3), the maximum diffraction-free distance *Z*_*b*_ can be calculated and the value is about 60 mm.

**Figure 6:**
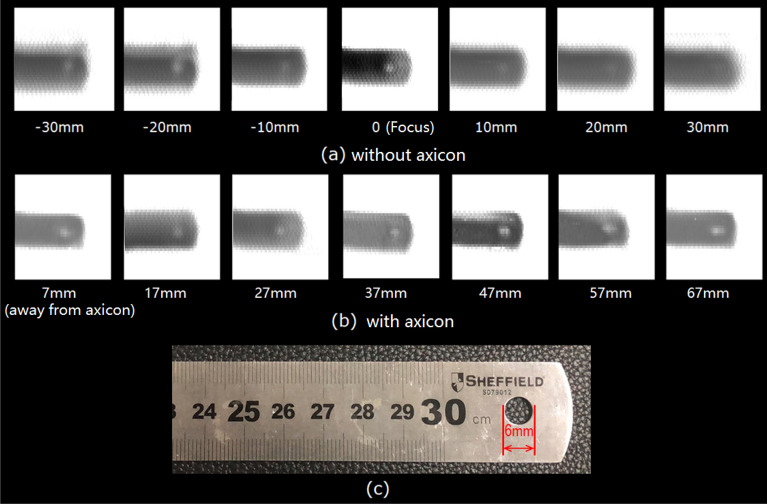
(**a**) The first group experiment results based on the system without axicon; (**b**) the second group experiment results based on the large focal depth imaging system; (**c**) photo of the sample.

To demonstrate the effectiveness of the proposed system, two groups of comparative experiments are carried out. The imaged target is a steel rule with a hole of 6 mm in diameter as shown in Fig. 6c. The first group experiment is based on the simplified system without axicon. The sample is placed at the system’s focus for imaging, and then it is moved forward and backward along the Z axis. The moving step is 10 mm. The results are shown in Fig. 6a. Among them, the subscript ‘0’ stands for the focus position, the subscript ‘− 10 mm’ represents the forward movement of 10 mm along the Z axis, and the subscript ‘10 mm’ represents the backward movement of 10 mm along the Z axis. It can be seen that the focal depth of the system is about 10 mm, beyond which the image becomes blurred and the 6 mm hole of the ruler cannot be clearly imaged. The second group experiment is based on large focal depth system with axicon. Above all, the sample is placed at the position of 7 mm behind the axicon. And then, it is moved back along the Z axis for imaging. The moving step is also 10 mm. The imaging results are shown in Fig. 6b. It can be seen that the focal depth of the system has been greatly improved. Whether it is at 7 mm or 67 mm, the ruler and its hole can be clearly imaged. Therefore, it shows that the focal depth can reach 60 mm.

**Figure 7:**
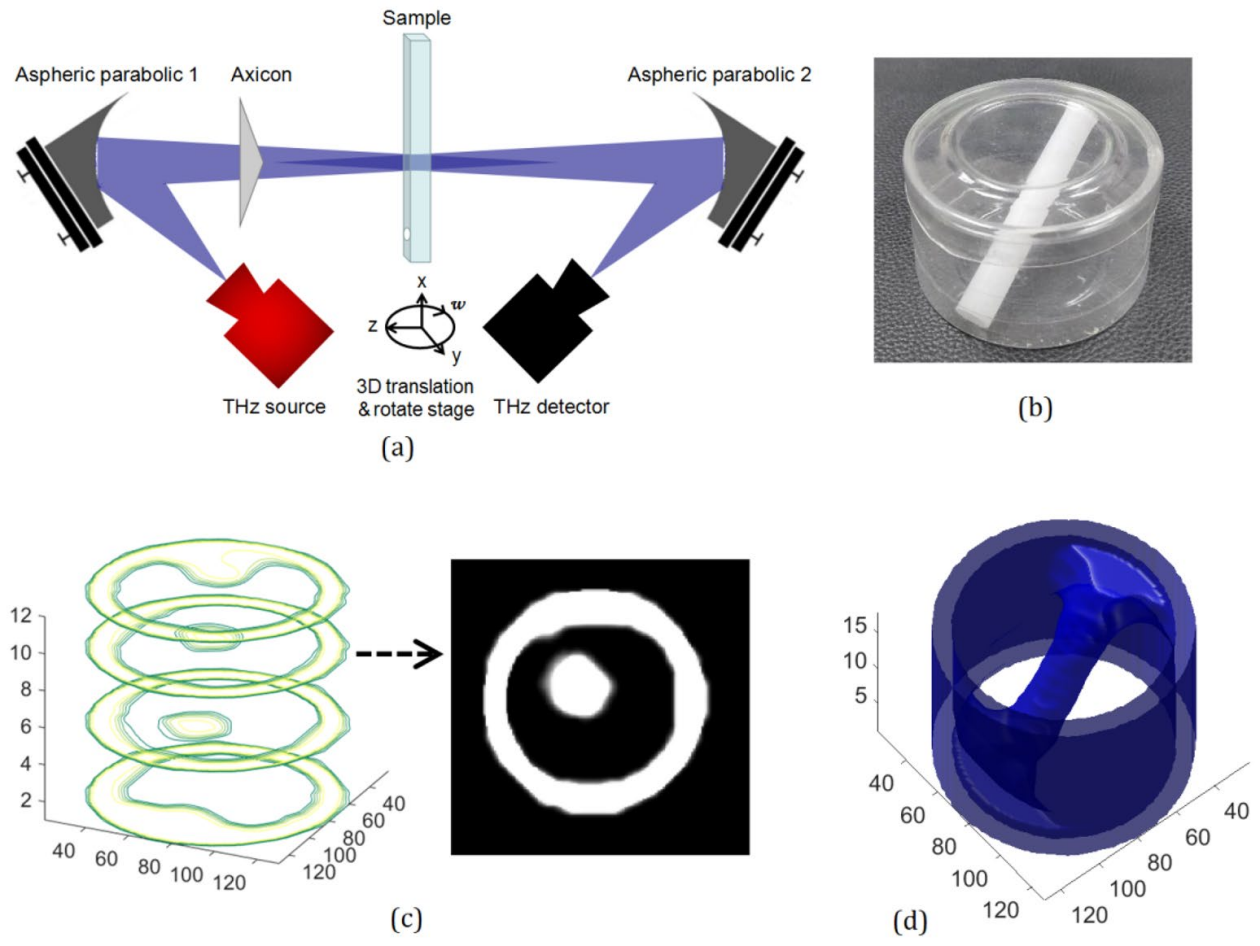
(**a**) Schematic of the 3D computed tomography system; (**b**) the target (a round plastic bottle with a diameter of 60 mm); (**c**) four layers (1th, 5th, 9th, 12th layers) of section images; (**d**) the reconstructed 3D image.

Next, the imaging system can be improved for 3D computed tomography (CT). Its schematic diagram is shown in Fig. 7a and the only improvement is the addition of a rotating control motor with x-axis as the rotation center. The steps for CT imaging can be described as follows: (1) rotate the target object through the *w* direction and acquire the transmission energy value every 3.6 degrees (360 degrees in total); (2) move the y-axis by a step of 2 mm, and then repeat the first step. When the y-axis moves 80 mm, the data of the first section of the object is obtained. (3) Move the x-axis by a step of 2 mm, and then repeat step 1 and step 2 to obtain the data of the second section of the object; (4) when the x-axis moves 32 mm, a total of 16 cross-section data are obtained; (5) the 16 real section images of object are recovered by filtering back projection algorithm^20^; (6) the section images are denoised and edge smoothed. Finally, the three-dimensional images of the object are obtained by reconstructing multiple sections. Furthermore, the steps for 3D reconstruction are as follows: (1) Image denoising by filtering algorithm; (2) Image threshold segmentation; (3) Obtain the contour of the section images and smooth it; (4) Arrange the processed 2D image data in sequence to construct 3D reconstruction matrix; (5) Interlayer interpolation is required due to certain spacing between scanning layers; (6) draw the 3D image using the 3D data matrix. Figure 7b shows the target which is a round plastic bottle (60 mm diameter and 35 mm height) with a plastic rod (9 mm diameter) in it. Figure 7c selects four layers (1th, 5th, 9th, 12th layers) of section images to illustrate the image superposition process. The right image in Fig. 7c is the 9th section image. The final reconstructed 3D image is shown in Fig. 7d. It can be seen that the system has an enough depth of focus to achieve high quality 3D imaging of the bottle.

## Conclusion

A simple off-axis large focal depth THz imaging system based on the special surface components is proposed and demonstrated. It includes only two aspherical parabolic mirrors and one axicon. To solve the problem of aspheric surface preparation, a new hybrid 3D printing method is proposed. Moreover, a geometrical theoretical model is proposed to calculate the maximum diffraction-free distance of axicon system in the case of oblique incidence. The experiments demonstrate that the focal depth can reach 60 mm, which is consistent with the design and theoretical calculation. Moreover, the system can be improved to a computed tomography system to realize 3D imaging. Consequently, we believe that the design, model and fabrication of 3D printed special surface components could serve as a promising and practical platform to advanced low-cost and high-performance large focal depth THz imaging for a wide range of applications.

## Method

**Figure 8:**
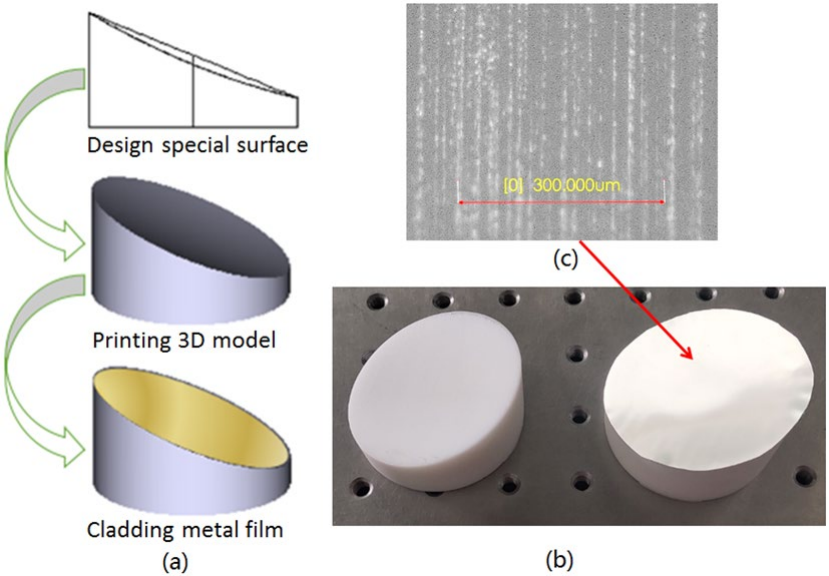
(**a**) Schematic of 3D printing and metal cladding method; (**b**) photos of fabricated parabolic mirrors: The left is 3D printed model and the right is final mirror with Al cladding; (**c**) microscopic image of the specific area on the surface.

Although the aspheric parabolic mirror has better focusing performance, it’s difficult to accurately process by traditional optical mirror processing technology. Considering the advantages of 3D printing technology, it is possible to use 3D printing to fabricate aspheric surfaces. In the paper a hybrid processing method for terahertz special surface components is proposed. Its brief flow chart is shown in Fig. 8a and detailed steps are as follows. Firstly, the parabolic mirror based on aspheric surface is designed and optimized by ZEMAX. Secondly, the 3D CAD model file of the designed parabolic mirror is exported. Thirdly, import the 3D model file to the slicing software of 3D printing machine, check the 3D structure and adjust structure’s position. Fourthly, set the printing accuracy and slice the model for printing. Fifthly, print the 3D model of the parabolic mirror according to the slicing file. Finally, cover the aluminum film (about 0.1 mm) on the parabolic mirror.

The printing accuracy of 3D printer is the main factor affecting the processing accuracy. In the paper the printer used is Ultimaker 2 extended+ . Its minimum layer thickness can reach 20 microns, the positioning accuracy of X and Y axis is 12.5 microns, and the positioning accuracy of Z axis is 5 microns. For special surfaces such as parabolic mirrors and axicons, the minimum layer thickness of the printer determines the accuracy, so the accuracy of the surface can be up to 20 microns.

The photos of the fabricated mirrors are presented in Fig. 8b: The left mirror is the 3D printed model; the right is the final aspherical parabolic mirror with metal cladding. The metallization process we adopted is to paste the 0.1 mm thick aluminum film directly onto the 3D printing component. Because the surface is aspheric while the aluminum film is flat and thick, some areas are not smooth. Finally, the quality of the aspherical surface is examined by a microscope, as shown in Fig. 8c. It can be seen that there are rough and uneven particles on the surface of the axicon, but the size is small (on the order of microns). Considering that the imaging wavelength of the system is about 3 mm (located in the millimeter wave and terahertz band), the roughness has little effect on the performance of the imaging system. The results of subsequent imaging experiments also confirmed this view. It should be mentioned that metallization (metal coating) of 3D printing components is an important and promising research field. In this paper, the lowest cost and lower precision metallization method is adopted. To improve the accuracy and ensure smoothness, two schemes can be adopted in the future: 1) electroless nickel plating, followed by copper plating; 2) direct sputtering of metal materials.
